# Effect of brain acidification on depression-related behaviors in diabetes mellitus

**DOI:** 10.3389/fpsyt.2023.1277097

**Published:** 2023-11-29

**Authors:** Yusuke Temma, Kisho Obi-Nagata, Yoshio Hoshiba, Ryuhei Miyake, Yuta Katayama, Hideo Hagihara, Norimitsu Suzuki, Tsuyoshi Miyakawa, Keiichi I. Nakayama, Akiko Hayashi-Takagi

**Affiliations:** ^1^Laboratory for Multi-Scale Biological Psychiatry, Center for Brain Science, RIKEN, Wako, Japan; ^2^Laboratory of Medical Neuroscience, Institute for Molecular and Cellular Regulation, Gunma University, Maebashi, Japan; ^3^Department of Psychiatry and Neuroscience, Graduate School of Medicine, Gunma University, Maebashi, Japan; ^4^Department of Integrative Physiology, Graduate School of Medicine, Gunma University, Maebashi, Japan; ^5^Department of Molecular and Cellular Biology, Medical Institute of Bioregulation, Kyushu University, Fukuoka, Japan; ^6^Division of Systems Medical Science, Center for Medical Science, Fujita Health University, Toyoake, Japan

**Keywords:** comorbidity, depression, diabetes mellitus, brain acidification, anhedonia

## Abstract

Major depressive disorder (depression) is a leading cause of disability. The severity of depression is affected by many factors, one of which being comorbidity with diabetes mellitus (DM). The comorbidity of depression with DM is a major public health concern due to the high incidence of both conditions and their mutually exacerbating pathophysiology. However, the mechanisms by which DM exacerbates depression remain largely unknown, and elucidating these regulatory mechanisms would contribute to a significant unmet clinical need. We generated a comorbid mouse model of depression and DM (comorbid model), which was extensively compared with depression and DM models. Depressive and anhedonic phenotypes were more severe in the comorbid model. We thus concluded that the comorbid model recapitulated exacerbated depression-related behaviors comorbid with DM in clinic. RNA sequencing analysis of prefrontal cortex tissue revealed that the brain pH homeostasis gene set was one of the most affected in the comorbid model. Furthermore, brain pH negatively correlated with anhedonia-related behaviors in the depression and comorbid models. By contrast, these correlations were not detected in DM or control group, neither of which had been exposed to chronic stress. This suggested that the addition of reduced brain pH to stress-exposed conditions had synergistic and aversive effects on anhedonic phenotypes. Because brain pH was strongly correlated with brain lactate level, which correlated with blood glucose levels, these findings highlight the therapeutic importance of glycemic control not only for DM, but also for psychiatric problems in patients with depression comorbid with DM.

## 1 Introduction

Depression, or major depressive disorder, is a common psychiatric disorder that affects more than 300 million people worldwide (1). Although the symptoms of depression are diverse and variable, depressive mood and anhedonia (loss of pleasure) are particularly emphasized in diagnostic manuals such as the Diagnostic and Statistical Manual of Mental Disorders (2), as these symptoms have debilitating effects on depression severity (3). In fact, according to the estimate of the World Health Organization (WHO), depression will be the single largest contributor to the burden of disease by 2030 (4). Furthermore, according to an extensive WHO survey of 60 countries, the prevalence of depression is higher in persons with chronic physical conditions (5). Comorbid depression is a serious public health concern in developed countries with aging demographics that increase medical symptom burden, functional impairment, and increased risk of morbidity and mortality (5–8). Indeed, depression comorbid with chronic physical disorders results in the worst health scores of all disease states (5). Physical disorders commonly comorbid with depression include diabetes mellitus (DM), cancer, autoimmune diseases, heart diseases, thyroid disorders, and chronic pain conditions (5, 9–12). Among comorbid conditions, DM is one of the leading exacerbating factors for depression (5, 13, 14). In fact, depression and DM are frequently comorbid (5, 15), and in individuals with DM, especially poorly controlled DM, complications worsen depression (15). Like depression, DM is also a common disorder, affecting over 400 million persons worldwide (16). Despite this robust epidemiological evidence and the impacts of DM and depression on health conditions, the interaction between these disorders remains largely unknown. DM induces inflammation, oxidative stress, and glycation stress (17–20), but the roles of these pathologies as exacerbating factors for depression remain elusive.

The difficulty in elucidating systemic interactions between DM and depression underscores the importance of holistic and data-driven approaches using genetically and environmentally well-controlled samples. Therefore, in the present study, we used an inbred mouse model to generate comorbid depression and DM. We utilized the chronic restraint stress (CRS) model of depression (21, 22), as chronic stress is a common clinical cause of depression, and this model causes typical depression-related symptoms such as chronic depressive and anhedonic phenotypes. To induce DM, we used streptozotocin (STZ), which induces insulin deficiency by damaging pancreatic beta cells (23, 24). A comorbid model (comorbid) was generated by subjecting animals to CRS 4 weeks after STZ administration. By comparing four groups, control, CRS, DM, and comorbid, with behavioral analyses. To gain mechanistic insight into the exacerbating effect of DM on depression, we focused on the prefrontal cortex (PFC), a brain region consistently impaired in human major depressive disorder (25), and performed histological and gene expression analyses, electrophysiology, and measurements of inflammatory factor levels.

## 2 Materials and methods

### 2.1 Ethical considerations

All mouse procedures were approved by the institutional animal care guidelines and the guidelines of the Animal Care Committee of the RIKEN Center for Brain Science and Gunma University.

### 2.2 Generation of mouse models

Mice were housed in a room maintained at a constant temperature (25°C) under a 12 h light/12 h dark cycle, and mice were provided free access to food and water. All mice used in the study were male and in the C57BL/6J background, and were purchased from Japan SLC, Inc. (Hamamatsu, Japan). DM was induced by STZ injection. STZ (Sigma-Aldrich, St. Louis, Missouri) was solubilized in sodium citrate buffer (pH 4.5) and 150 mg/kg body weight STZ was injected intraperitoneally into each mouse at the age of 60 days. Two weeks after STZ injection, mice with blood glucose levels <300 mg/dL were considered not to fulfil the requirement for inclusion in the DM model and were excluded from further experiments. A depression model was induced by subjecting mice to the CRS protocol. Mice in the CRS group were placed in a well-ventilated 50 mL polypropylene conical tube (352070, Corning Inc., Corning, New York), and a striped paper towel (Kim Towel folding in four, 380 × 330 mm; Nippon Paper Crecia Co., Ltd., Tokyo, Japan) was placed in the tube to fill the space between the mouse and the tube cap. Restraint was performed for 6 h on 21 consecutive days, 4 weeks after vehicle or STZ injection.

### 2.3 Blood glucose and HbA1c measurement

Levels of blood glucose and HbA1c were measured using samples collected from the tail vein. Tail vein blood sampling was performed with a 1 mm single-use lancet (Goldenrod Animal Lancet, Medipoint, Mineola, New York). Blood glucose was measured using a glucose analyzer (Glutest Mint, Sanwa Chemical Co., Nagoya, Japan) and HbA1c levels were measured by DCA 2000 HbA1c immunoassay with the DCA Vantage Analyzer (Siemens Healthcare, Erlangen, Germany).

### 2.4 Quantification of fecal corticosterone

**Figure 1:**
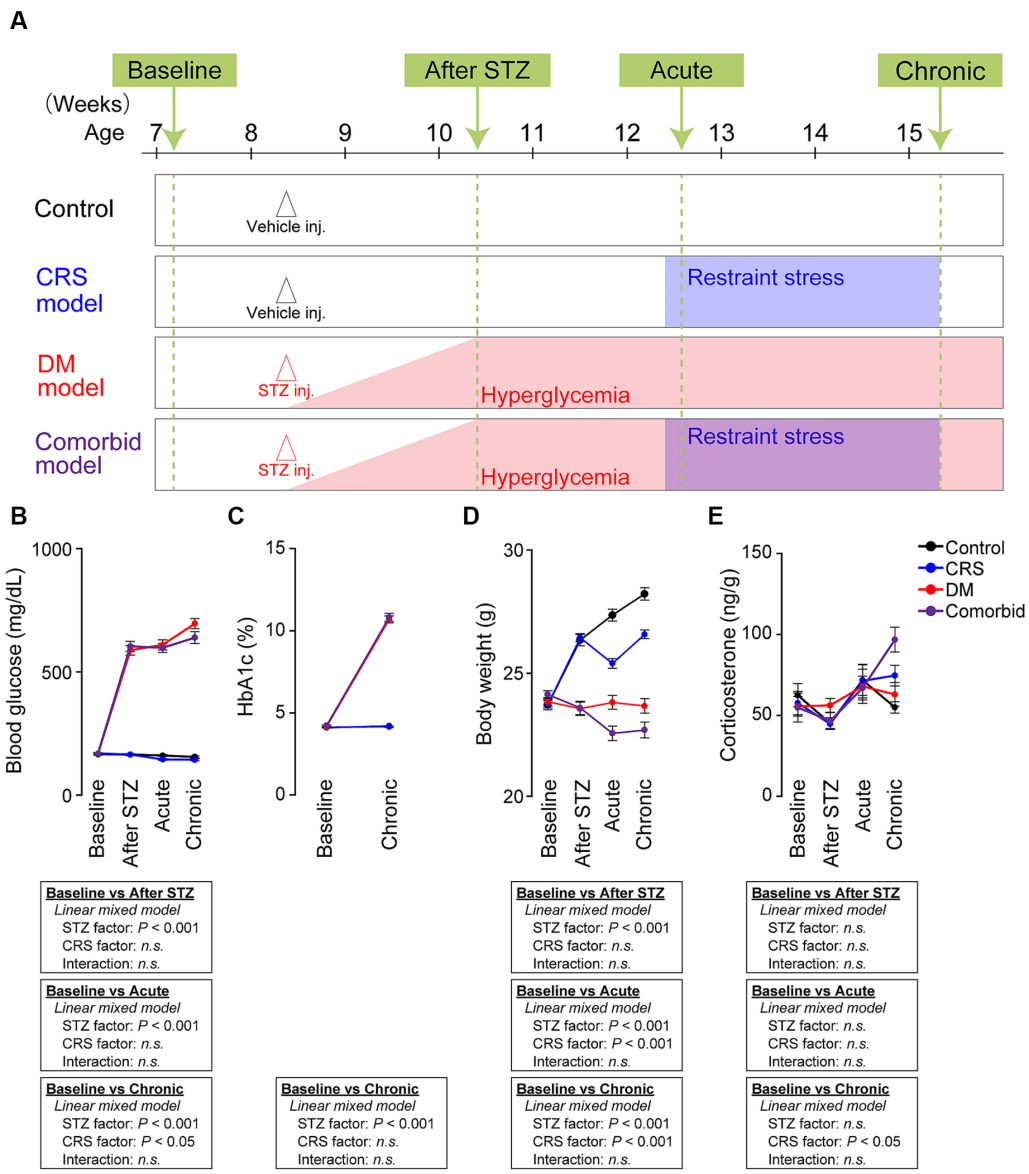
Establishment of a comorbid mouse model of depression and diabetes mellitus. **(A)** Experimental schedule of generating four conditions: control, CRS, DM, and comorbid. “Baseline” refers to the time point before vehicle or STZ injection. “After STZ” indicates 4 weeks after injection of STZ or vehicle. “Acute” indicates the first 3 days following the onset of restraint stress. “Chronic” refers to the last 3 days of CRS. Mice were subjected to either vehicle or STZ injection at the age of 60 days to generate control or DM mice. Mice in the DM and comorbid group exhibited hyperglycemia (blood glucose >300 mg/dL) from 1 week after STZ injection (red shade). Mice in the CRS and comorbid groups were subjected to CRS for 21 days beginning 4 weeks after Vehicle or STZ injection (blue shade). **(B)** Change in blood glucose of the four models. Control group, number of mice (*n*) = 54; CRS group, *n* = 57; DM group, *n* = 62; comorbid group, *n* = 57. **(C)** HbA1c change of the four treatment groups. Control group, *n* = 28; CRS group, *n* = 30; DM group, *n* = 34; comorbid group, *n* = 30. **(D)** Changes in body weight of the four treatment groups. Control group, *n* = 54; CRS group, *n* = 57; DM group, *n* = 62; comorbid group: *n* = 57. **(E)** Change in fecal corticosterone level of the four experimental groups. Control group, *n* = 17–21; CRS group, *n* = 17–21; DM group, *n* = 17–18; comorbid group, *n* = 12–15; n.s., not significant. Data are expressed as mean ± SEM.

Quantification of fecal corticosterone was performed as described previously (26). Feces from mice maintained in individual cages were collected at four time points: baseline, after STZ, acute CRS, and chronic CRS (Figure 1A). Strip litter (Pulsoft, Oriental Yeast Co.; Pulmas μ, Material Research Center Co., Kawasaki, Japan) was used to ensure effective collection of feces, which were stored at −80°C until analysis. Fecal samples were completely dried, weighed, and thoroughly ground. One milliliter aqueous ethanol [ethanol: deionized distilled water (DDW), 8:2 (v/v)] was added to 50 mg fecal powder, boiled (99°C) for 5 min, and vortexed for 10 min. The boiling and vortexing steps were repeated two additional times. After centrifugation (10 min at 2,500 × g), the supernatant was transferred into another tube and stored at −20°C until performing the ELISA assay. We used a Corticosterone Enzyme Immunoassay Kit (Arbor Assays, Ann Arbor, Michigan) according to the manufacturer’s protocol. An aliquot of 2.5 μL supernatant was added to one well of the ELISA plate per animal.

### 2.5 Behavioral analysis

#### 2.5.1 Measurement of activity in home cage

Single-housed mice in their home cages were individually placed in the chamber of an activity sensor system (O’Hara & Co., Ltd., Tokyo, Japan). Food and water were available *ad libitum*. The amount of activity was recorded every 30 min except during times of food intake measurement, feces collection, mouse restraint and release, drug administration, blood collection, and behavioral tests.

#### 2.5.2 Measurement of wheel running activity

Mice were individually housed in cages (24 cm wide × 11 cm deep × 14 cm high) equipped with a running wheel (5 cm wide × 14 cm diameter). LD 12:12 h cycles (lights on at 8:00 local time) were controlled by a PC computer system (O’Hara & Co., Ltd.). Light was provided by white LED and the intensity was 20–50 lux at the level of mouse’s eyes in the cage. Food and water were available *ad libitum*. Wheel running activity was recorded with an online PC computer system (O’Hara & Co., Ltd.) every minute except during times of feces collection, mouse restraint and release, drug administration, blood collection, and behavioral tests.

#### 2.5.3 Delayed and anticipatory activity index

Delayed and anticipatory activity index was measured as described previously (27). Briefly, the delayed activity index is defined as the amount of activity from the start of the light period to the start of the restraint procedure divided by the amount of activity during the dark period of the previous day. The anticipatory activity index is the amount of activity from the time the restraint is released to the end of the light period divided by the amount of activity in the dark period immediately after.

#### 2.5.4 Female urine sniffing test

A female urine sniffing test was performed as described previously (28, 29). Mice were singly housed in freshly made home cages for a habituation period of 10 min under illumination of 30 lx. Subsequently, two plain cotton tips were secured on the center of the cage wall, and mice were allowed to sniff and habituate to the tips for a period of 30 min. Then, the tips were removed and replaced by two tips infused with fresh female mouse oestrus urine or with fresh male mouse urine. These applicators were presented and secured at the two sides of the cage wall simultaneously. Sniffing time for each tip was scored by a trained observer for a period of 3 min. Female preference was calculated using the following formula: time spent sniffing female urine/(time spent sniffing female urine + time spent sniffing male urine).

#### 2.5.5 Female encounter test

The female encounter test was performed as described previously (30). Briefly, a test mouse was placed in the central chamber of an opaque acrylic-modified polyvinyl chloride box (50 cm × 50 cm × 30 cm) divided into three interconnected chambers under illumination of 100 lx. The clear partitions (30 cm × 30 cm) had openings that allow the animal to move freely from one chamber to another. After a 90 min habituation period, unfamiliar sexually naïve C57B/6J male and female mice were introduced into the intruder boxes (8 cm × 6.5 cm × 30 cm). The test and intruder mice were allowed to interact through the wire-mesh walls for 15 min, and the intruder mice were then removed. The amount of time spent around male and female zones was measured to estimate the behavioral reactivity of mice to the intruder for the final 10 min. To simplify the indices of motivation, preference to female encounter was also calculated as a percentage score for each intruder: preference (%) = (time spent in female zone/total time spent in male and female zones during 10 min period of measurement) × 100.

#### 2.5.6 Forced swim test

For the forced swim test, the apparatus consisted of four Plexiglas cylinders (20 cm in height and 10 cm in diameter, O’Hara & Co., Ltd.) filled with dilute sodium hypochlorite solution at 23 ± 1°C up to a height of 7.5 cm, which was illuminated at 400 lx. At 16 weeks of age, each male mouse was placed in the cylinders, and immobility time was recorded over a 10 min test period. Images were acquired at a frame rate of 2 Hz, and the mouse location and movement was measured by comparing the pixel signal intensity of each pair of successive frames (31). Immobility lasting <10 s was not included in analysis. Immobility time during the last 9 min of the test was used for comparison. Data acquisition and analysis were performed automatically using a TimeFZ2 video-imaging and analyzing system (O’Hara & Co., Ltd.).

#### 2.5.7 Open-field test

A test mouse was placed in the corner of an open-field apparatus (50 × 50 × 30 cm), which was illuminated at 100 lx. Total distance travelled was recorded and analyzed using LimeLight (ACTIMETRICS, Wilmette, IL) for 30 min.

#### 2.5.8 Novel object recognition test

The novel object recognition test was performed during 6 consecutive days, and three objects (Object A, B, and C) were prepared. On the first 4 days, mice were habituated to hands and to the empty arena (50 × 50 × 30 cm) at 100 lx for 6 min. On the fifth day, two identical objects (Object A) were placed 22.5 cm apart in the corner of the arena, and mice were allowed to freely explore the cage and objects for 15 min. After exploration, the mice were immediately returned to their home cages for 5 min, and the arena and objects ware cleaned with dilute sodium hypochlorite solution to avoid pheromonal cues. After that, the mouse was placed in the same chamber containing two different objects (Object A and B) for 5 min and was allowed to explore the objects to measure short-term memory retention. On the sixth day, the mouse was placed in the same chamber containing two different objects (Object A and C) for 15 min and allowed to explore the objects to measure long-term memory retention. The duration of mouse exploration behavior exhibited was determined using LimeLight (ACTIMETRICS, Wilmette, IL). The discrimination index was calculated using the following formula: (time exploring the novel object – time exploring the familiar object)/(time exploring the novel object + time exploring the familiar object).

#### 2.5.9 Y-maze test

Spatial working memory was measured using a Y-maze apparatus (three identical arms A, B, and C. Arm length: 40 cm, arm bottom width: 3 cm, arm upper width: 13 cm, height of wall: 15 cm, O’Hara & Co., Ltd.), which was illuminated at 100 lx. Each mouse was placed in the central area, and the number of entries into the arms were recorded for 10 min with a TimeYM2 video-imaging and analyzing system (O’Hara & Co., Ltd.). Spontaneous alternation behavior was defined as entry into all three arms on consecutive three entries (e.g., ABC, ACB, BAC, BCA, CAB, and CBA), and was calculated using the following formula: [number of spontaneous alterations/(total number of arm entries – 2)] × 100. To ensure that the behavioral analyses did not interfere with each other, we prepared five distinct cohorts, namely Cohort #1, female urine sniff test on day one, followed by the female encounter test on day two, and the forced swim test on day three; Cohort #2, female encounter test on day one, then the forced swim test on day two; Cohort #3, novel object recognition test spanning days one to six; Cohort #4, open field test on day one, and the Y-maze test on day two; Cohort #5, measurement of activity in the home cage was conducted first, followed by the open field test on day one, and the forced swim test on day two. Furthermore, a separate cohort was dedicated exclusively to measuring wheel running to preclude any interference with the remaining tests.

### 2.6 RNA sequencing and gene set enrichment analysis

Total RNA was extracted from the PFC brain region of male mice at 16 weeks of age using a TRIzol Plus RNA Purification Kit (Life Technology, Carlsbad, California). RNA-seq was performed as previously described (32). Complementary DNA was sequenced using a HiSeq 1500 system (Illumina, San Diego, California). The total amount of each transcript was calculated by a series of programs including TopHat2 (version 2.1.1) and Cufflinks (version 2.1.1). RNA-seq reads were mapped against the mouse (mm10) genome. GSEA was performed as described previously (33) using GSEA v.3.0.^1^ The gene set collections C5 (ontology gene set, 15937 gene sets) were obtained from Molecular Signature Database (MsigDB version 6.2; Broad Institute, http://www.broadinstitute.org/gsea/msigdb). Synergy scores were calculated using the following formula: min [NES (comorbid) – NES (CRS), NES (comorbid) – NES (DM)].

### 2.7 Measurement of pH

Mice were sacrificed via cervical dislocation and decapitation. Whole brains were immediately removed, frozen in liquid nitrogen and stored at −80°C until analysis. Brain pH was measured as previously described (34, 35). Briefly, mouse brains were homogenized using a tissue homogenizer equipped with a conical pestle in ice-cold distilled H_2_O (5 mL per 500 mg of tissue), and pH of the resulting homogenate was measured in triplicate using a pH meter (LAQUA F-72, Horiba Scientific, Kyoto, Japan).

### 2.8 Measurement of lactate

Brain lactate level was measured as previously described (34). Briefly, the concentration of lactate in brain homogenates was determined using a multi-assay analyzer (GM7 MicroStat; Analox Instruments, London, United Kingdom) according to manufacturer’s instructions.

### 2.9 Electrophysiological recordings

C57BL/6J mice at P136–P145 were deeply anesthetized with isoflurane at 2% and perfused with ice-cold NMDG aCSF oxygenated with 95% O_2_, 5% CO_2_ (92 mM NMDG, 2.5 mM KCl, 1.25 mM NaH_2_PO_4_, 30 mM NaHCO_3_, 20 mM HEPES, 25 mM glucose, 2 mM thiourea, 5 mM ascorbic acid, 3 mM Na-pyruvate, 0.5 mM CaCl_2_ and 10 mM MgCl_2_), as described previously (36, 37). After decapitation, brains were quickly removed and transferred into ice-cold oxygenated NMDG aCSF. Coronal slices were cut from the prefrontal cortex (300 μm) with a Leica VT1200S vibratome (Wezlar, Germany). Slices were incubated in NMDG aCSF at 35°C for 10 min and then transferred into a recovery chamber containing HEPES with aCSF (92 mM NaCl, 2.5 mM KCl, 1.25 mM NaH_2_PO_4_, 30 mM NaHCO_3_, 20 mM HEPES, 25 mM glucose, 2 mM thiourea, 5 mM ascorbic acid, 3 mM Na-pyruvate, 2 mM CaCl_2_, and 2 mM MgCl_2_) for 1 h at room temperature. Whole-cell patch-clamp recordings were performed from layer II/III pyramidal neurons in mPFC using a MultiClamp 700B (Molecular Devices, San Jose, CA). All recordings were performed at 33°C–35°C using pipettes with a resistance of 5–7 MΩ filled with K-based internal solution [130 mM K-gluconate, 10 mM KCl, 10 mM HEPES, 0.1 mM EGTA, 10 mM Na_2_-phosphocreatine, 4 mM Mg-ATP, 0.3 mM Na_2_-GTP, and 0.05 mM Alexa Fluor 488 (pH 7.25), 0.4% Biocytin]. Patch pipettes (BF150-86-10; Sutter Instrument, Novato, CA) were pulled with a pipette puller P1000 (Sutter Instrument, Novato, CA) and fire-polished using a microforge (MF-900; Narishige, Tokyo, Japan). The external aCSF solution contained 125 mM NaCl, 2.5 mM KCl, 26 mM NaHCO_3_, 1.25 mM NaH_2_PO_4_, 1 mM MgCl_2_, 2 mM CaCl_2_, and 25 mM glucose. Signals from current-clamp recordings were sampled at 50 kHz and filtered at 10 kHz. All data were acquired using AxoGraph X (AxoGraph, Sydney, Australia) with the NI USB-6363 interface (National Instruments, Austin, TX). Action potentials were measured by injecting a current for 1,000 ms in a stepwise manner from −120 to 640 pA in 40 pA increments. During recording, bridge balance was monitored. The resting membrane potential of a neuron was obtained under the current clamp (*I* = 0 pA), and the threshold for action potential generation was determined by the first derivative of the voltage reaching 20 V/s. Action potential parameters were measured from the first action potential evoked at a given threshold. All analyses were performed using AxoGraph X and Igor Pro 8.04 (WaveMetrics Inc., Lake Oswego, Oregon).

### 2.10 Visualization of dendritic morphology

Acute slices of the PFC were prepared, and whole-cell patch-clamp recordings were performed from layer II/III pyramidal cells to visualize dendrite and spine morphology. Potassium-based internal solution supplemented with 0.4% biocytin (B4261, Sigma-Aldrich, St. Louis, Missouri) was used. After recordings, slices containing biocytin-filled neurons were fixed overnight with 4% PFA. Fixed slices were permeabilized with 2.5% normal goat serum (v/v) in PBS with 0.3% Triton X-100 (v/v) for 1 h at room temperature and subsequently incubated overnight with streptavidin-Alexa Fluor 488 (1:1000; S32354, Invitrogen, Waltham, Massachusetts) in PBS with 2.5% normal goat serum (v/v) and 0.3% Triton X-100 (v/v). Confocal microscopy was performed as described below. The apical and basal dendrites of layers II/III pyramidal cells in the prelimbic and frontal association cortices were imaged.

### 2.11 Confocal imaging

Confocal imaging was performed using an inverted microscope (IX81; Olympus, Tokyo, Japan) and an oil-immersion objective lens (UplanSApo, 60×, 1.35 numerical aperture). The excitation wavelength was 473 nm for imaging Alexa Fluor 488. Images were acquired at 1× digital zoom, 1024 × 1024 pixels, 2 ms/pixel dwelling time, and *z*-axis step size 0.37 μm.

### 2.12 Analysis of dendrite morphology

Computer-based cell tracing software Neurolucida 360 (MBF Bioscience, Williston, Vermont) was used for 3D reconstruction of neurons. Neuro Explorer (MBF Bioscience) was used to measure dendrite length and for Sholl and spine density analyses. Total dendrite length was calculated as the sum of dendritic length from one neuron. Sholl analysis was used to assess the complexity of neural dendrites by placing 3D concentric circles in 20 μm increments starting at 10 μm from the soma, and the number of dendritic intersections with each circle was counted. Spine density was calculated from the number of spines divided by the length of the dendrite. Spines with a head-to-neck ratio >1.1 were considered to have a neck. Spines with head diameter >0.35 μm were classified as mushrooms, and otherwise were classified as thin. Spines with length-to-head ratio <2.5 were classified as stubby, and otherwise were labeled as thin. Spines >3 μm height were classified as filopodia.

### 2.13 Bio-Plex analysis

The concentration of cytokines and chemokines was measured in serum of facial vein blood, which was collected from anesthetized mice with a 5 mm single-use lancet (Goldenrod Animal Lancet, Medipoint, Mineola, New York). Dripping blood was directly collected from the puncture site and incubated at room temperature for 30 min. Samples were centrifuged for 50 min at 1000 × g at 4°C and serum stored at −80°C. A Bio-Plex mouse cytokine and chemokine assay (Bio-Rad, Hercules, California) was used for simultaneous quantitation of tumor necrosis factor (TNF)-α, granulocyte colony-stimulating factor (G-CSF), granulocyte macrophage colony-stimulating factor (GM-CSF), interleukin (IL)-1a, IL-2, IL-3, IL-4, IL-5, IL-6, IL-9, IL-10, IL-12 (p40), IL-12 (p70), IL-13, IL-17, interferon (IFN)-γ, eotaxin, keratinocyte-derived chemokine (KC), macrophage inflammatory protein (MIP)-1a, MIP-1b, regulated upon activation normal T cell expressed and secreted (RANTES), and monocyte chemoattractant protein (MCP)-1.

### 2.14 Statistical analyses

Statistical analyses were conducted using EZR software (38). Data were expressed as mean ± SEM unless otherwise specified. The differences between the four groups were analyzed by two-way analyses of variance (ANOVA). If there was a statistically significant interaction effect, a *post hoc* Tukey’s multiple-comparisons test was performed. Differences between the CRS and comorbid group were analyzed by unpaired *t*-test. Differences between the Sholl profiles of four groups were analyzed by a linear mixed model. Relationships between pH and depression-related behavior were analyzed using Pearson’s correlation. Differences between the CRS group and the non-CRS group were analyzed using analyses of covariance (ANCOVA). The statistics are summarized in Supplementary Table S1.

## 3 Results

### 3.1 Generation of a new mouse model of comorbid depression and DM

We first aimed to establish a comorbid model of depression and DM. We used the CRS exposure protocol as a depression model because CRS causes morphological changes in the nervous system and depressive-like behaviors in rodents (21). Among a variety of DM models (23), we selected STZ injection model, which is a well-established DM model. STZ damages pancreatic beta cells to induce insulin deficiency and hyperglycemia (24). In our experimental schedule (Figure 1A), we first administered STZ to 8 weeks-old mice to generate the DM model. To determine if the STZ-injected mice exhibited diabetic features and to exclude animals that did not fulfill DM criteria (see Methods section for details), we measured blood glucose and hemoglobin (Hb)A1c. STZ-injected mice that passed the exclusion criteria were considered DM animals, and exhibited significantly higher blood glucose (Figure 1B) and HbA1c (Figure 1C). Four weeks after injection of vehicle or STZ, half of the mice were subjected to CRS, in which animals were restrained in a 50 mL conical tube for 6 h daily over 21 consecutive days. Body weight was significantly decreased by both STZ injection and restraint stress (Figure 1D), which is consistent with the clinical phenotypes of depression and DM. We also measured fecal corticosterone, the primary murine glucocorticoid hormone. CRS significantly increased the fecal corticosterone level in the chronic phase, but not in the acute phase (Figure 1E). As chronic and persistent increases in glucocorticoid hormones, although not specific, can potentially serve as a biomarkers of depression (39), suggesting that CRS-induced chronic mild stress evoked the depression-relevant state. Taken together, these findings demonstrated that the comorbid model recapitulated the biological characteristics of depression and DM.

### 3.2 Exacerbated depression-related behaviors in the comorbid model

**Figure 2:**
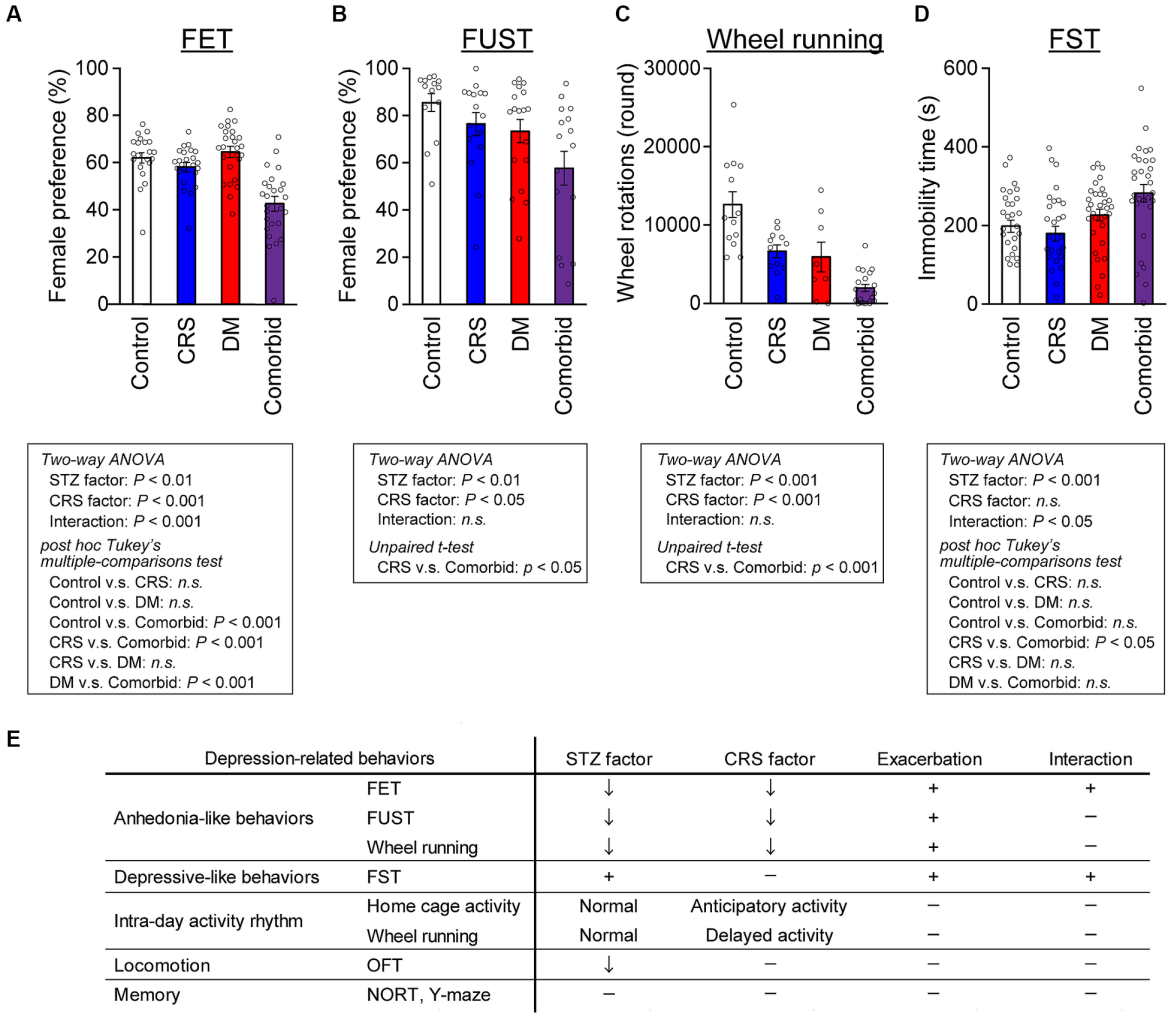
Exacerbation of depression-related behaviors in comorbid mice **(A)** Female preference measured by FET. Control group, *n* = 21; CRS group, *n* = 20; DM group, *n* = 23; comorbid group, *n* = 23. **(B)** Female preference measured by FUST. Control group, *n* = 14; CRS group, *n* = 16; DM group, *n* = 18; comorbid group, *n* = 16. **(C)** Wheel running activity level of mice in the chronic phase. Control group, *n* = 12; CRS group, *n* = 12; DM group, *n* = 9; comorbid group, *n* = 12. **(D)** Immobility time in the FST. Control group, *n* = 26; CRS group, *n* = 26; DM group, *n* = 32; comorbid group, *n* = 30; n.s., not significant. Data are expressed as mean ± SEM. **(E)** Summary of behavioral analyses. The down arrow in the STZ and CRS columns indicates that diabetes or CRS significantly evoked the indicated depression-related behaviors. The plus marks in the exacerbation and interaction columns denote that more-severe depression-related behavior was detected in the comorbid model than in the CRS, and that diabetes and CRS-induced depression interacted.

We subsequently performed behavioral analyses of the four models in the chronic phase or after the CRS phase, especially with the aim of distinguishing between two distinct axes characteristic of depression: depressive mood-related and anhedonia-related behaviors (Figure 2). A typical behavioral measure that can be used to access depressed mood is the forced swim test (FST). Anhedonia-related behaviors, such as sexual behaviors, are reward-seeking, and highly and naturally motivated behaviors. These are assessed using the female encounter test (FET) and the female urine sniffing test (FUST), with low scores considered to be an index of anhedonia (28, 30). FET preference was lower in the comorbid group than in the control, CRS, or DM groups, and an interaction between STZ administration and CRS was detected in a two-way ANOVA, suggesting that the decreased FET preference in the comorbid group was exacerbated by the combination of DM and CRS conditions (Figure 2A). In the FUST, both STZ injection and CRS significantly decreased female preference (Figure 2B). Voluntary wheel running is also rewarding for mice because it satisfies the instinctive need for exercise and exploration (40). Both STZ treatment and CRS exposure significantly decreased wheel running activity (Figure 2C). Direct comparison of CRS and comorbid groups (Figures 2B,C) demonstrated that the phenotype was significantly exacerbated in the comorbid group relative to the CRS group, consistent with exacerbation of anhedonic behaviors in this group. The forced swim test (FST), which is used to assess depressive-like behaviors, revealed that the immobility time was significantly longer in the comorbid group than in the CRS group (Figure 2D).

We subsequently measured locomotor activity in the standard home cage and voluntary wheel running in wheel-equipped cages over the entire experimental period, because this is a non-invasive assessment suitable for tracking circadian rhythms and longitudinal changes in behaviors over whole periods (Supplementary Figure S1A). Both STZ treatment and CRS exposure significantly decreased home cage activity in the chronic phase (Supplementary Figure S1B). To quantify potential circadian patterns, we measured the delayed activity index and anticipatory activity index, which suggest delayed and anticipatory circadian rhythms, respectively (27). While STZ treatment did not affect activity patterns in either the home cage or the wheel-equipped cage (Supplementary Figures S1C–F), CRS significantly increased the anticipatory activity index in the home cage (Supplementary Figure S1D) and delayed activity in the wheel-equipped cage (Supplementary Figure S1E), suggesting that CRS disturbed the circadian rhythm, which is a depressive symptom. To assess other behavioral aspects of each model, additional assessments such as short- and long-term memory, and spatial working memory were performed, none of which differed between the four groups (Supplementary Figures S1H–J). Locomotor activity was only decreased in the open-field test (OFT) in the STZ-treated groups (Supplementary Figure S1G). Taken together, these behavioral analyses indicated that comorbid mice exhibited specific exacerbation of depression-related behaviors (Figure 2E), while higher cognitive functions such as spatial working memory and spatial reference memory were largely intact.

### 3.3 Disruption of brain pH homeostasis and relationship with depression-related behaviors

**Figure 3:**
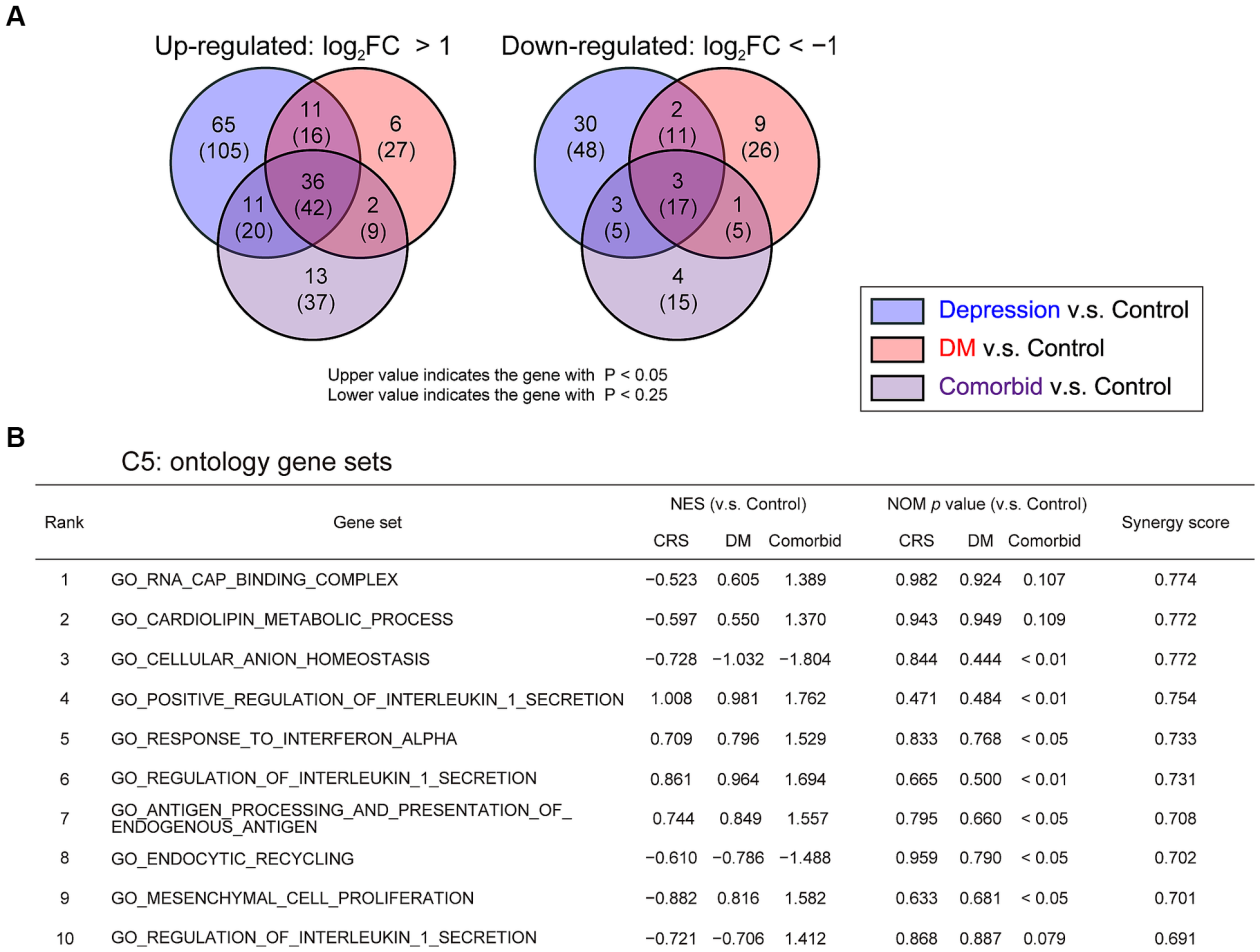
Change in pH homeostasis-related gene sets and activation of inflammation-related gene sets in comorbid mice **(A)** Venn graph illustrating the overlap in PFC genes. Log_2_ FC >1 for upregulated genes or log_2_ FC <−1 for downregulated genes, respectively. **(B)** GSEA of differentially regulated genes for gene set collection C5 in the four experimental groups. Control group, *n* = 3; CRS group, *n* = 3; DM group, *n* = 3; comorbid group, *n* = 3. NES, normalized enrichment score; NOM, normal.

**Figure 4:**
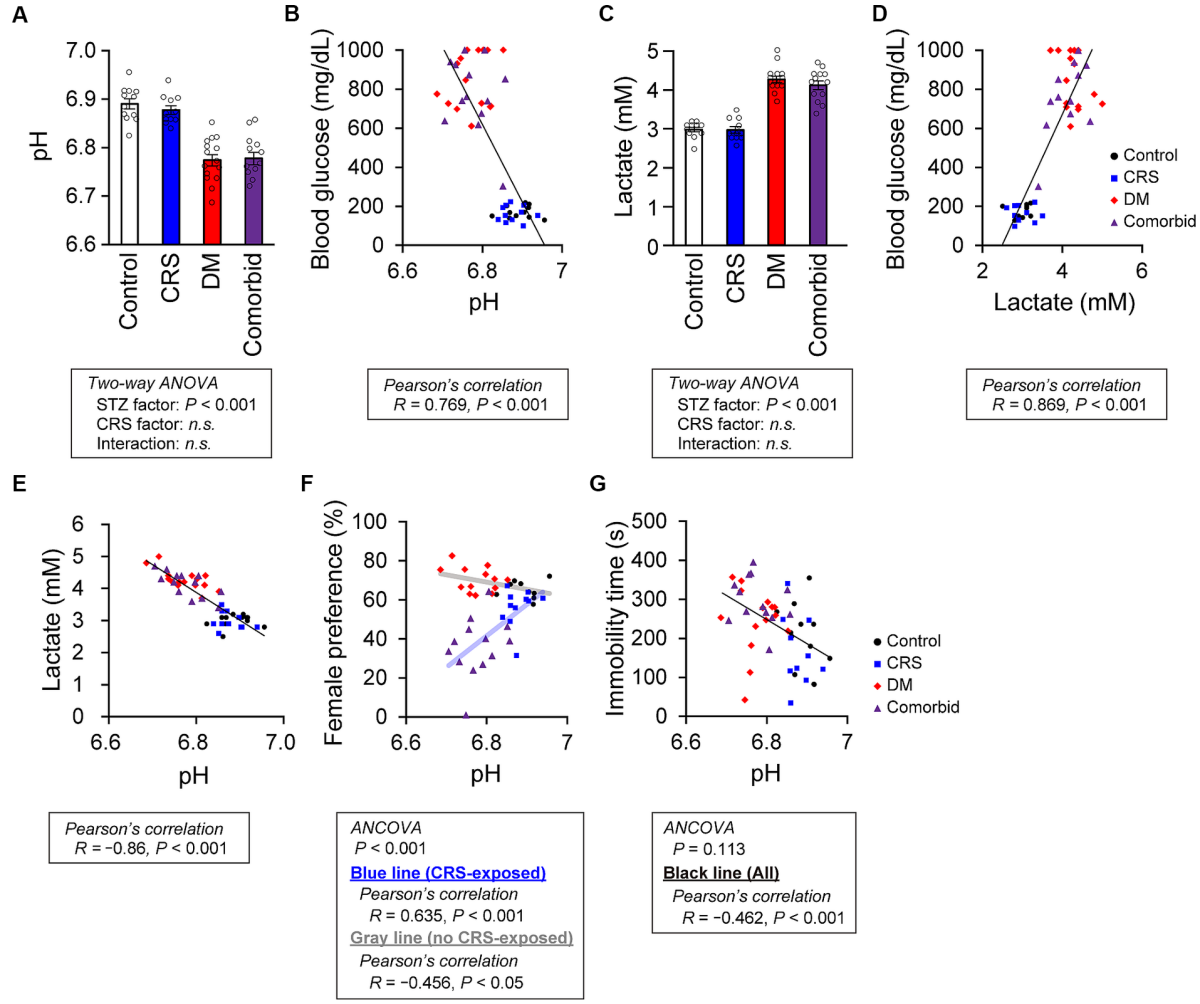
Relationship between brain pH level and depression-related behaviors. **(A)** pH of postmortem mouse brains. Control group, *n* = 12; CRS group, *n* = 11; DM group, *n* = 15; comorbid group, *n* = 13. **(B)** Relationship between brain pH and blood glucose level. A black line indicates the fitted line for all plots. Control group: *n* = 12, CRS group: *n* = 11, DM group: *n* = 15, comorbid group: *n* = 13. **(C)** Brain lactate level. Control group, *n* = 12; CRS group, *n* = 11; DM group, *n* = 15; comorbid group, *n* = 13. **(D)** Relationship between brain lactate and blood glucose level. A black line indicates the fitted line for all plots. Control group, *n* = 12; CRS group, *n* = 11; DM group, *n* = 15; comorbid group, *n* = 13. **(E)** Relationship between the brain lactate and brain pH. A black line indicates the fitted line for all plots. Control group, *n* = 12; CRS group, *n* = 11; DM group, *n* = 15; comorbid group, *n* = 13. **(F)** Relationship between brain pH and female preference score in the FET. A light-blue line and a black line indicate the fitted lines for plots of the CRS-exposed group (CRS and comorbid groups) or non-exposed group (control and DM groups), respectively. Control group, *n* = 9; CRS group, *n* = 11; DM group, *n* = 13; comorbid group: *n* = 13. **(G)** Relationship between immobility time in the FST and brain pH. A black line indicates the fitted line for plots of all groups. Control group, *n* = 12; CRS group, *n* = 10; DM group, *n* = 15; comorbid group, *n* = 13. Data are expressed as mean ± SEM.

To take an unbiased approach towards identifying new molecular mechanisms for mutual DM-depression exacerbation, mRNA sequencing analysis was performed on PFC tissue. Thirteen genes were upregulated (log_2_ fold change >1), and four genes were downregulated (log_2_ fold change <−1) specifically in the comorbid group (Figure 3A and Supplementary Table S2). To determine which biological processes were altered in the comorbid model, we conducted gene set enrichment analysis (GSEA), identifying that a gene set involving brain pH homeostasis and an interferon regulation gene set were two of the most affected gene sets in the comorbid group (Figure 3B). To validate these findings, we first measured serum cytokine and chemokine levels. While the levels of many cytokine and chemokine factors were significantly different in the STZ-injected group or CRS group (Supplementary Figure S2), the levels of none of these factors were altered in the comorbid group, and none of these factors exhibited an interaction between CRS and STZ injection. This may be because peripheral and brain cytokine levels do not always correlate (41, 42). We therefore shifted our focus to brain pH homeostasis. Measurement of brain pH revealed that STZ injection robustly decreased brain pH and increased brain lactate level (Figures 4A,C). Blood glucose, brain lactate levels, and brain pH were strongly intercorrelated (Figures 4B,D,E), suggesting that the decrease in brain pH was due to hyperglycemia.

To determine how decreased brain pH could exacerbate depression-related behaviors, we first examined the neuronal properties of each group. Other than decreased thin spine density on apical dendrites in the STZ group (Supplementary Figure S3A), no significant difference in spine density (Supplementary Figures S3A,B), spine size (Supplementary Figures S3C,D), or dendritic structures (Supplementary Figure S4) were detected between groups. Electrophysiological analysis of PFC neurons revealed that only action potential threshold was significantly different in the STZ-injected group (Supplementary Figure S5). Because the involvement of cellular processes in depression-related behaviors were unclear, we directly examined the relationship between brain pH and depression-related behaviors. First, we performed analysis of covariance (ANCOVA) to analyze the relationship between brain pH and behavioral parameters, while controlling for the effects of CRS as a covariate. CRS significantly affected FET but not FST (Figures 4F,G), so subsequent analyses were divided into CRS-exposed and non-exposed groups only for FET. Brain pH was negatively correlated with female preference specifically in the CRS-exposed group but not in the non-CRS-exposed group (Figure 4F). This suggested that the decrease in female preference, a highly motivated reward in male mice, was not due to a non-specific physical effect such as general fatigue caused by DM, as decreased female preference occurred only when CRS and DM conditions were combined. Furthermore, brain pH significantly correlated with immobility time in the FST and was not specific to the comorbid group (Figure 4G).

## 4 Discussion

We here established a comorbid mouse model of DM and depression, which exhibited more-severe depression-related symptoms (Figure 2). This is the first study to identify that depression-related behaviors are exacerbated by diabetes in mouse models of depression. Notably, CRS did not increase immobility time in the FST, which is a typical phenotype of depression mouse models (Figure 2D). We considered that our CRS model was a mild depression model or a model of subthreshold depression (43). Nevertheless, the fact that co-occurrence of DM evoked more-severe depression-related symptoms in a mild depression model further underscores the synergistic effects of the comorbidities of DM and CRS-induced depression. Therefore, we contend that the newly developed comorbid model successfully recapitulated the aversive interaction of DM with depression-related behaviors. GSEA findings in this model implied that disruption of brain pH homeostasis was associated with exacerbation of depression-related behaviors (Figure 3). While both DM and comorbid mice exhibited lower brain pH (Figure 4A), only comorbid mice exhibited loss of female preference in the FET (Figure 2E). This was further supported by the finding that brain pH was significantly correlated with female preference specifically in the CRS-exposed group but not in the non-CRS-exposed group (Figure 4F). These results suggested that the combination of CRS and decreased brain pH synergistically contributed to anhedonia-like behaviors. To our knowledge, this is the first report suggesting that decreased brain pH could exacerbate depression-related behaviors.

The effect of pH on exacerbation of depression-related behaviors remains unclear. Previous studies demonstrated that brain pH decrease inhibits AMPA receptor currents (44), increases generation of reactive oxygen species (45), and activates the acid-sensing ion channel 1a (46), and that these changes may contribute to the pathophysiology of depression models (29, 47, 48). Previous studies have also demonstrated that decreased AMPA receptor currents are related to spine size (49) and stability (50). In addition, spine density is decreased in PFC pyramidal neurons of rats with STZ-induced diabetes (51). Therefore, we examined whether morphological changes occurred under low pH conditions in our models. Morphological analysis of PFC dendritic spines revealed that only the density of thin spines on apical dendrites was decreased (Supplementary Figure S2A). A prior aging study reported that thin spine density of PFC neurons decreases with age in rhesus monkeys (52). These results suggest that the STZ-induced morphological changes in synaptic plasticity have some similarities to those associated with aging.

The primary limitation of our study is that we did not determine causality between brain pH and depression-related behaviors. However, in the social defeat stress model, another model of depression, multiple regression analyses suggest that increased brain lactate levels are preferentially correlated with decreased social interaction and increased anxiety-like behaviors (53). Further investigations of the relationship between brain acidification and behavior using different models of depression are needed. Likewise, we used only one model of DM. The STZ injection model of insulin deficiency is a common type I DM model. On the other hand, type II DM involves insulin resistance, and a recent study suggested that some common pathophysiological mediators are associated with insulin resistance and mental conditions (54, 55). Therefore, future studies are needed to determine if depression-related behaviors are affected in type II DM models, or are affected by decreased brain pH in this context. Also, we did not detect hyperglycemia in CRS-exposed mice (Figure 1B). Previous studies demonstrated that in the KM mouse, which is outbred, CRS exposure induces hyperglycemia (56) and that in some mice exposed to chronic social defeat stress, the blood glucose level is increased (57). These studies suggested that other depression models could be informative in investigating the mechanisms of co-exacerbation of diabetes and depression.

A recent study suggested that decreased brain pH is a common endophenotype in schizophrenia patients, bipolar disorder patients, and mouse models of multiple psychiatric disorders (34). Our results suggested that lower brain pH may be an important endophenotype for depression as well. Furthermore, manipulation of brain pH is a potential novel treatment strategy for depression-related conditions in DM patients. In daily clinical practice, controlling blood glucose is critical for therapeutic management not only of DM, but also psychiatric problems in patients with depression comorbid with DM.

## Data availability statement

The original contributions presented in the study are publicly available. This data can be found here: BioProject PRJDB16335 (https://www.ncbi.nlm.nih.gov/bioproject/?term=PRJDB16335), DDBJ Sequence Read Archive DRA016854 (https://ddbj.nig.ac.jp/resource/sra-submission/DRA016854) and Genome expression Archive E-GEAD-634 (https://ddbj.nig.ac.jp/public/ddbj_database/gea/experiment/E-GEAD-000/E-GEAD-634/).

## Ethics statement

The animal study was approved by the Animal Care Committee of the RIKEN Center for Brain Science and Gunma University. The study was conducted in accordance with the local legislation and institutional requirements.

## Author contributions

YT: Data curation, Formal analysis, Investigation, Methodology, Validation, Visualization, Writing – original draft, Writing – review & editing. KO-N: Conceptualization, Data curation, Formal analysis, Investigation, Methodology, Project administration, Supervision, Writing – review & editing. YH: Formal analysis, Funding acquisition, Investigation, Writing – review & editing. RM: Formal analysis, Investigation, Writing – review & editing. YK: Formal analysis, Investigation, Methodology, Writing – review & editing. HH: Formal analysis, Investigation, Writing – review & editing. NS: Data curation, Formal analysis, Investigation, Writing – review & editing. TM: Supervision, Writing – review & editing. KN: Supervision, Writing – review & editing. AH-T: Conceptualization, Funding acquisition, Project administration, Supervision, Visualization, Writing – original draft, Writing – review & editing.

## Footnotes

^1^ http://www.broadinstitute.org/gsea
