# Supplementary material for: Effect of brain acidification on depression-related behaviors in diabetes mellitus
Source: Front Psychiatry. 2023 Nov 29;14:1277097. doi: 10.3389/fpsyt.2023.1277097 (PMC10716456; doi:10.3389/fpsyt.2023.1277097)
Supplement: Supplementary file 1 [file Table_1.DOCX]

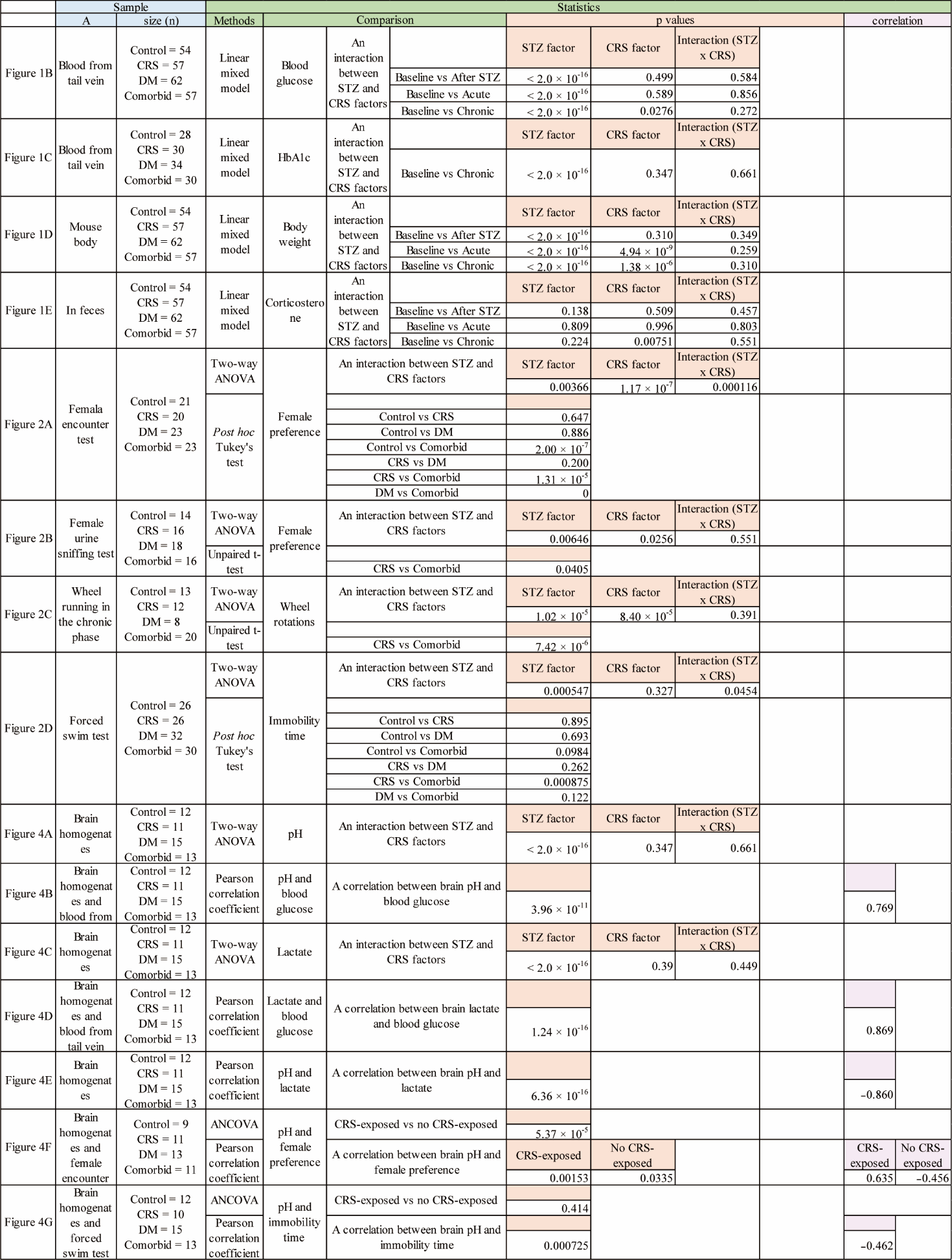
Table S1 (Continued) for measures and statistics.


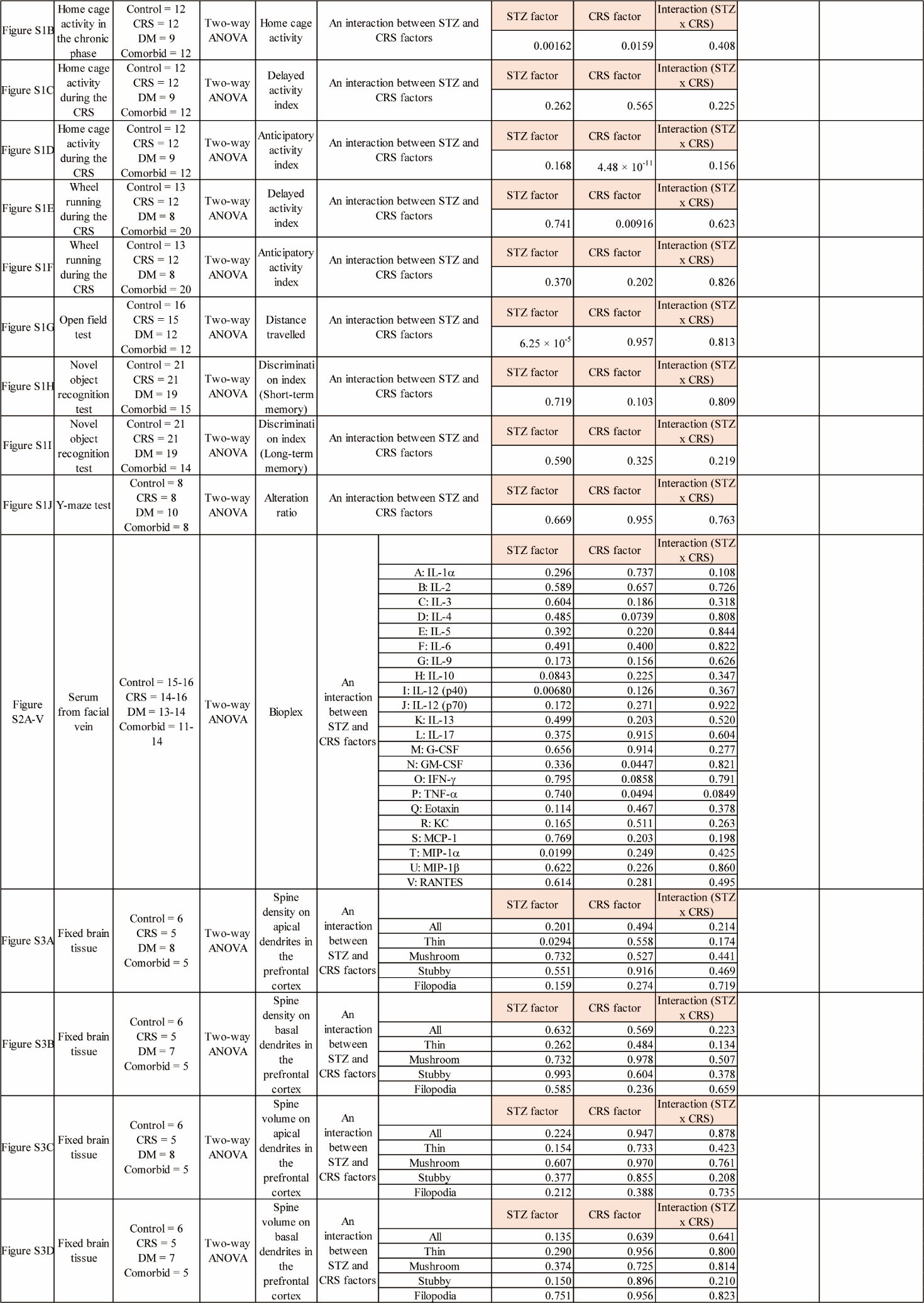


Table S1 (Continued) for measures and statistics.


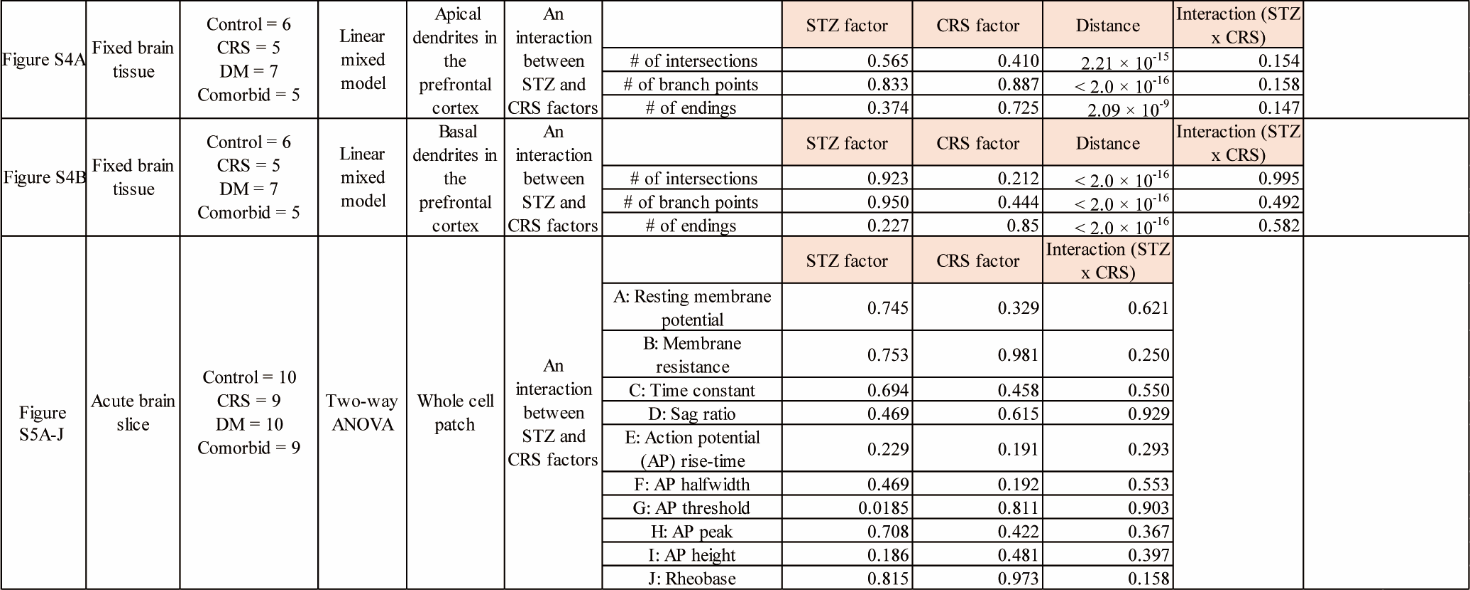


Table S1 for measures and statistics.
