# Supplementary material for: Effect of brain acidification on depression-related behaviors in diabetes mellitus
Source: Front Psychiatry. 2023 Nov 29;14:1277097. doi: 10.3389/fpsyt.2023.1277097 (PMC10716456; doi:10.3389/fpsyt.2023.1277097)
Supplement: Supplementary file 2 [file Table_2.DOCX]

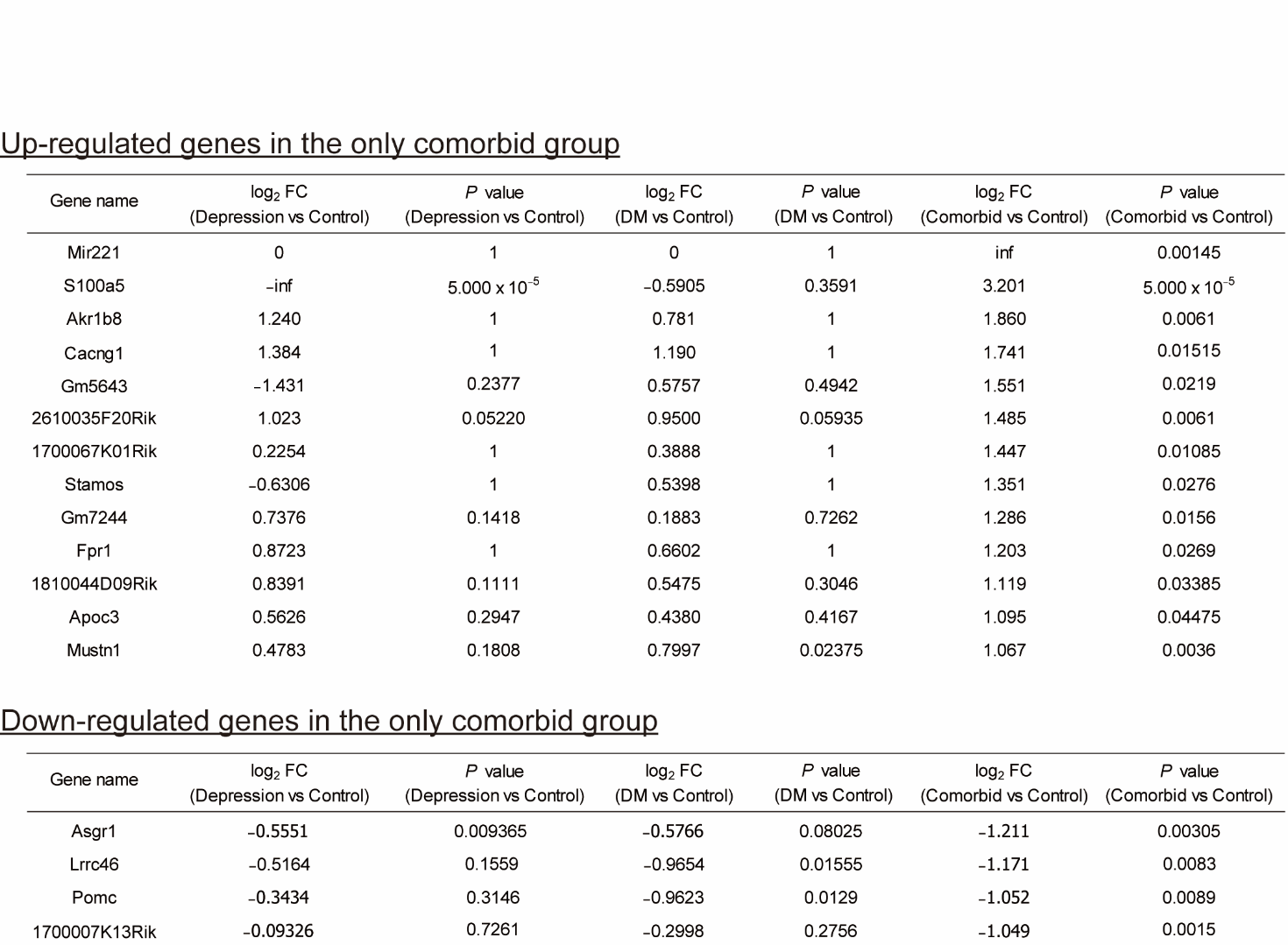


Table S2.

**(Upper)**

The 13 genes that were up-regulated only in the comorbid group.

Mir221: microRNA 221

S100a5: S100 calcium binding protein A5

Akr1b8: aldo-keto reductase family 1 member B8

Cacng1: calcium channel voltage-dependent gamma subunit 1

Gm5643: predicted gene 5643

2610035F20Rik: RIKEN cDNA 2610035F20 gene

1700067K01Rik: RIKEN cDNA 1700067K01 gene

Stamos: signal transducing adaptor molecule 1 opposite strand

Gm7244: predicted gene 7244

Fpr1: formyl peptide receptor 1

1810044D09Rik: RIKEN cDNA 1810044D09 gene

Apoc3: apolipoprotein C-III

Mustn1: musculoskeletal embryonic nuclear protein 1

**(Lower)** The 4 genes that were down-regulated only in the comorbid group.

Asgr1: asialoglycoprotein receptor 1

Lrrc46: leucine rich repeat containing 46

Pomc: pro-opiomelanocortin-alpha

1700007K13Rik: RIKEN cDNA 1700007K13 gene
