# Supplementary material for: Effect of brain acidification on depression-related behaviors in diabetes mellitus
Source: Front Psychiatry. 2023 Nov 29;14:1277097. doi: 10.3389/fpsyt.2023.1277097 (PMC10716456; doi:10.3389/fpsyt.2023.1277097)
Supplement: Supplementary file 3 [file Data_Sheet_1.PDF]

Supplementary figure 1

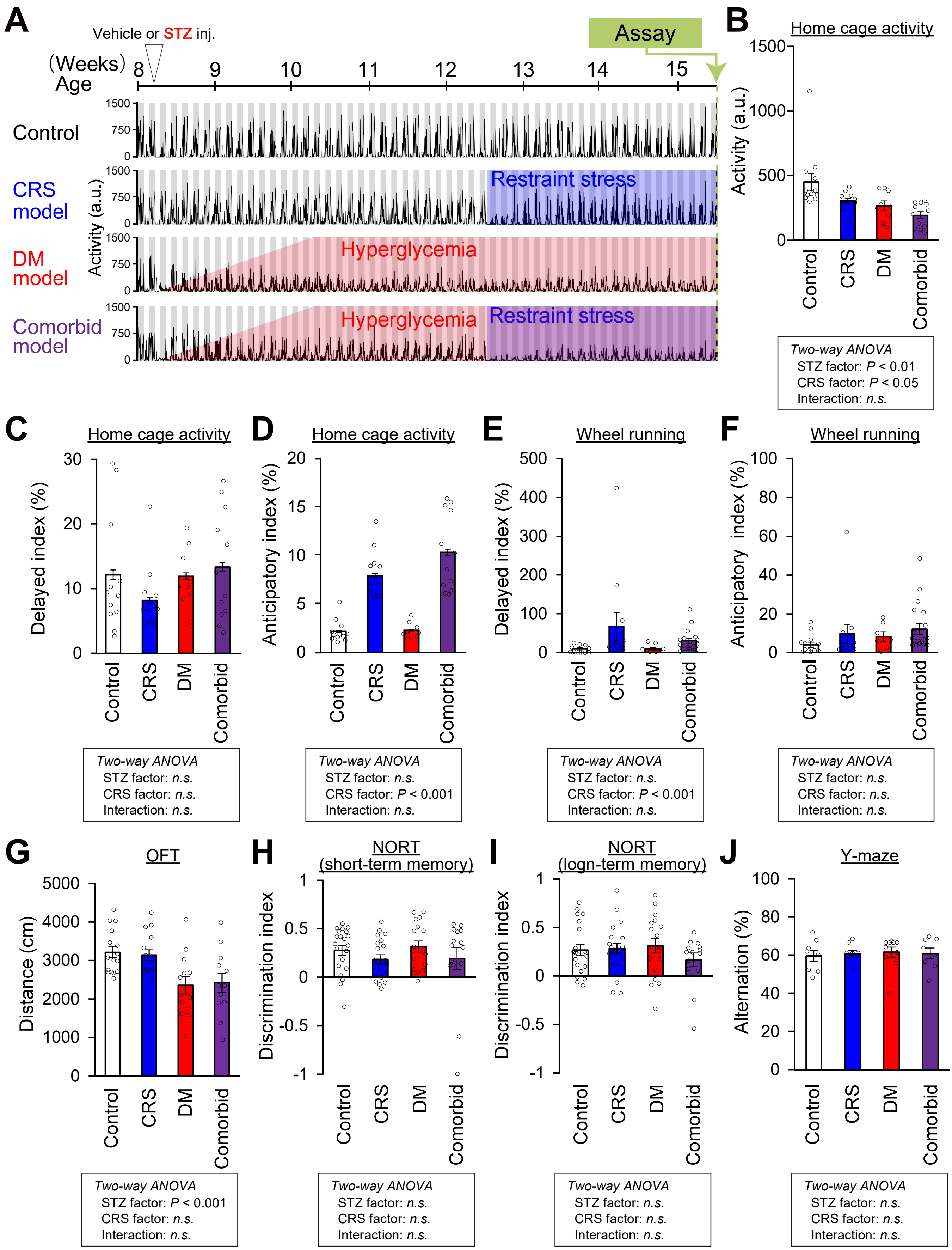

Supplementary figure 1: Results of behavioral tests in the four models. **(A)** Change in home cage activity throughout the entire study periods. Gray and white periods indicate the dark and light periods, respectively. **(B)** Amount of home cage activity during the chronic phase. Control group, n = 13; CRS group, n = 12; DM group, n = 8; Comorbid group, n = 20. **(C)** Home cage delayed activity index during the CRS exposure period. Control group, n = 13; CRS group, n = 12; DM group, n = 8; Comorbid group: n = 20. **(D)** Home cage anticipatory activity index during the CRS exposure period. Control group, n = 13; CRS group, n = 12; DM group, n = 8; Comorbid group, n = 20. **(E, F)** Delayed (E) or Anticipatory (F) activity index of wheel running during the CRS exposure period. Control group, n = 12; CRS group, n = 12; DM group, n = 9; Comorbid group, n = 12. **(G)** Total distance travelled for the OFT. Control group, n = 16; CRS group, n = 15; DM group, n = 13; Comorbid group, n = 12. **(H, I)** Discrimination index for **(H)** short- and **(I)** long-term memory in the novel object recognition test (NORT). Control group, n = 21; CRS group, n = 21; DM group, n = 18; Comorbid group, n = 15. **(J)** Alteration rate in the Y-maze test in the baseline and chronic phase. Data are expressed as means ± SEM. Control group, n = 8; CRS group, n = 8; DM group, n = 10; Comorbid group, n = 8; *n.s.*, not significant.

Supplementary figure 2

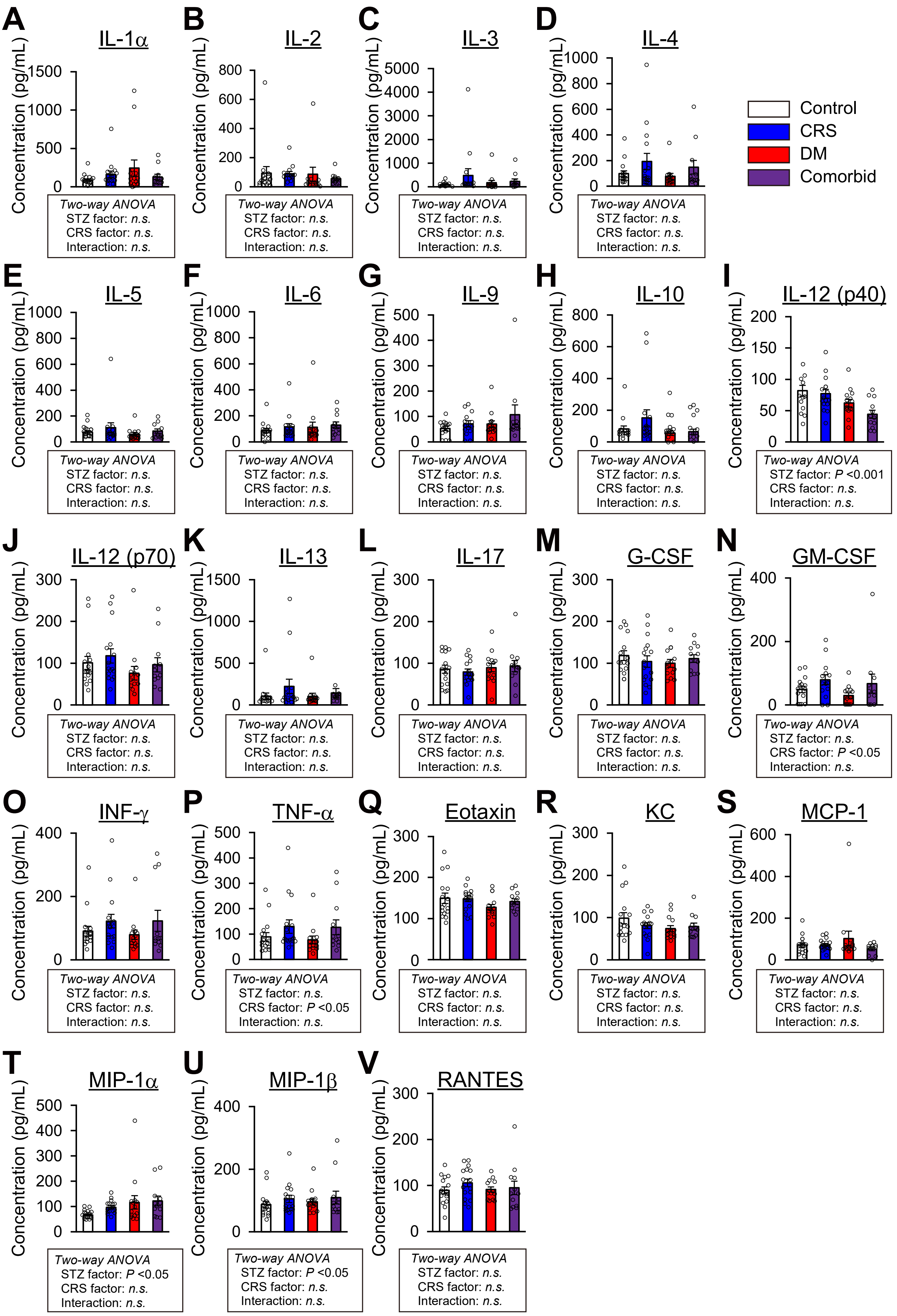

Supplementary figure 2 Bio-Plex assay analysis of serum cytokines and chemokines. **(A-P)** Cytokines and **(Q-V)** chemokines in serum samples from mice of the four experimental groups. Data are expressed as means ± SEM. Control group, n = 15–16; CRS group, n = 14–16; DM group, n = 13–14; Comorbid group, n = 11–14; *n.s.*, not significant; IL, interleukin; TNF, tumor necrosis factor; G-CSF, granulocyte colony-stimulating factor; GM-CSF, granulocyte macrophage colony-stimulating factor; IFN, interferon; KC, keratinocyte-derived chemokines; MCP, monocyte chemotactic protein; MIP, macrophage inflammatory protein; RANTES, regulated on activation, normal T cell expressed and secreted.

Supplementary figure 3

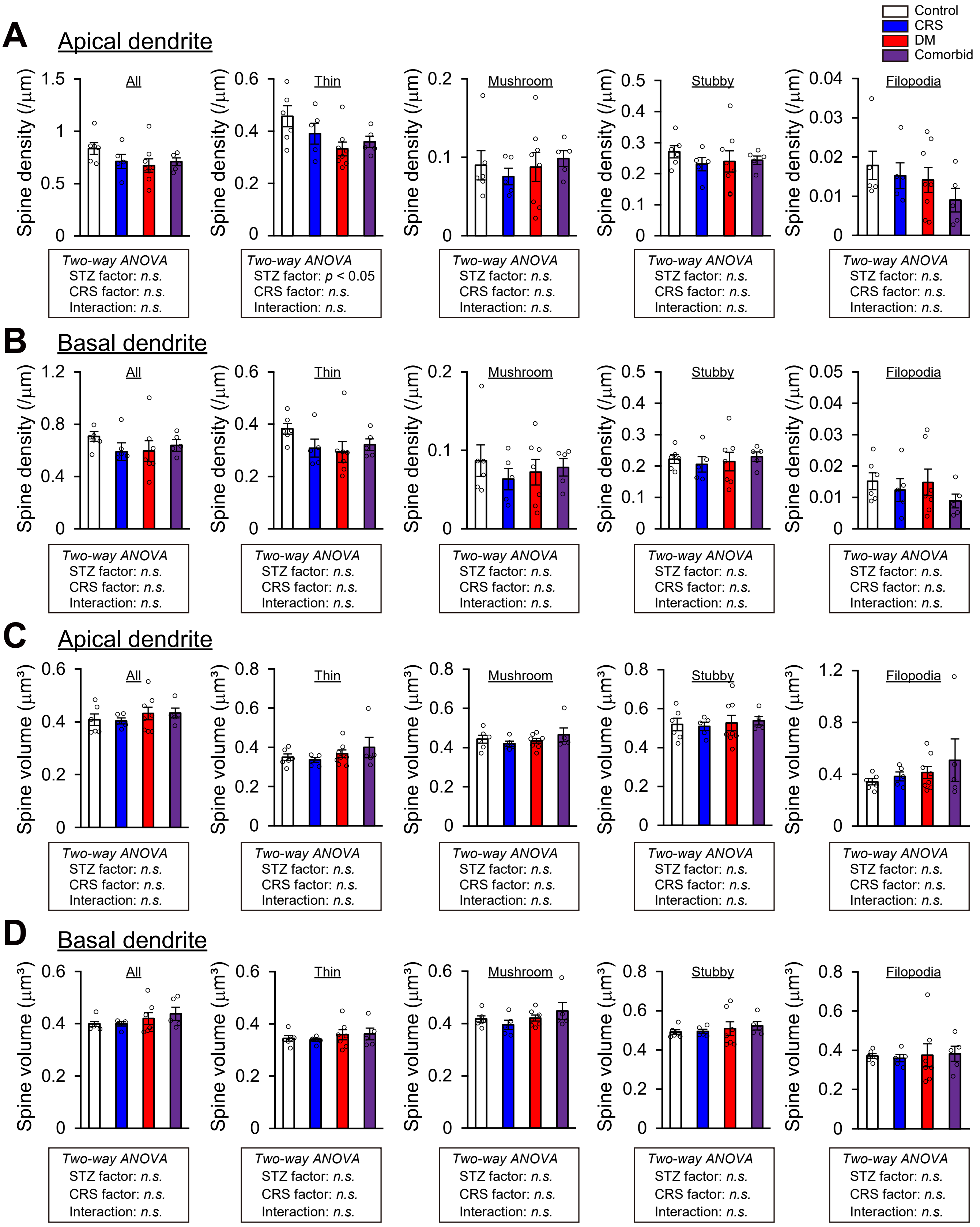

Supplementary figure 3 Analysis of dendritic spine density and size on apical and basal dendrites. Spines on apical dendrites of mouse PFC neurons were morphologically classified into five categories: all spine, thin spine, mushroom spine, stubby spine, and filopodia. **(A)** Spine density on apical dendrites. Control group, n = 6; CRS group, n = 5; DM group, n = 8; Comorbid group, n = 5. **(B)** Spine density on basal dendrites. Control group, n = 6; CRS group, n = 5; DM group, n = 8; Comorbid group, n = 5. **(C)** Spine volume on apical dendrites. Control group, n = 6; CRS group, n = 5; DM group, n = 7; Comorbid group, n = 5. **(D)** Spine volume on basal dendrites. Control group, n = 6; CRS group, n = 5; DM group, n = 7; Comorbid group, n = 5; *n.s.*, not significant. Data are expressed as mean ± SEM.

Supplementary figure 4

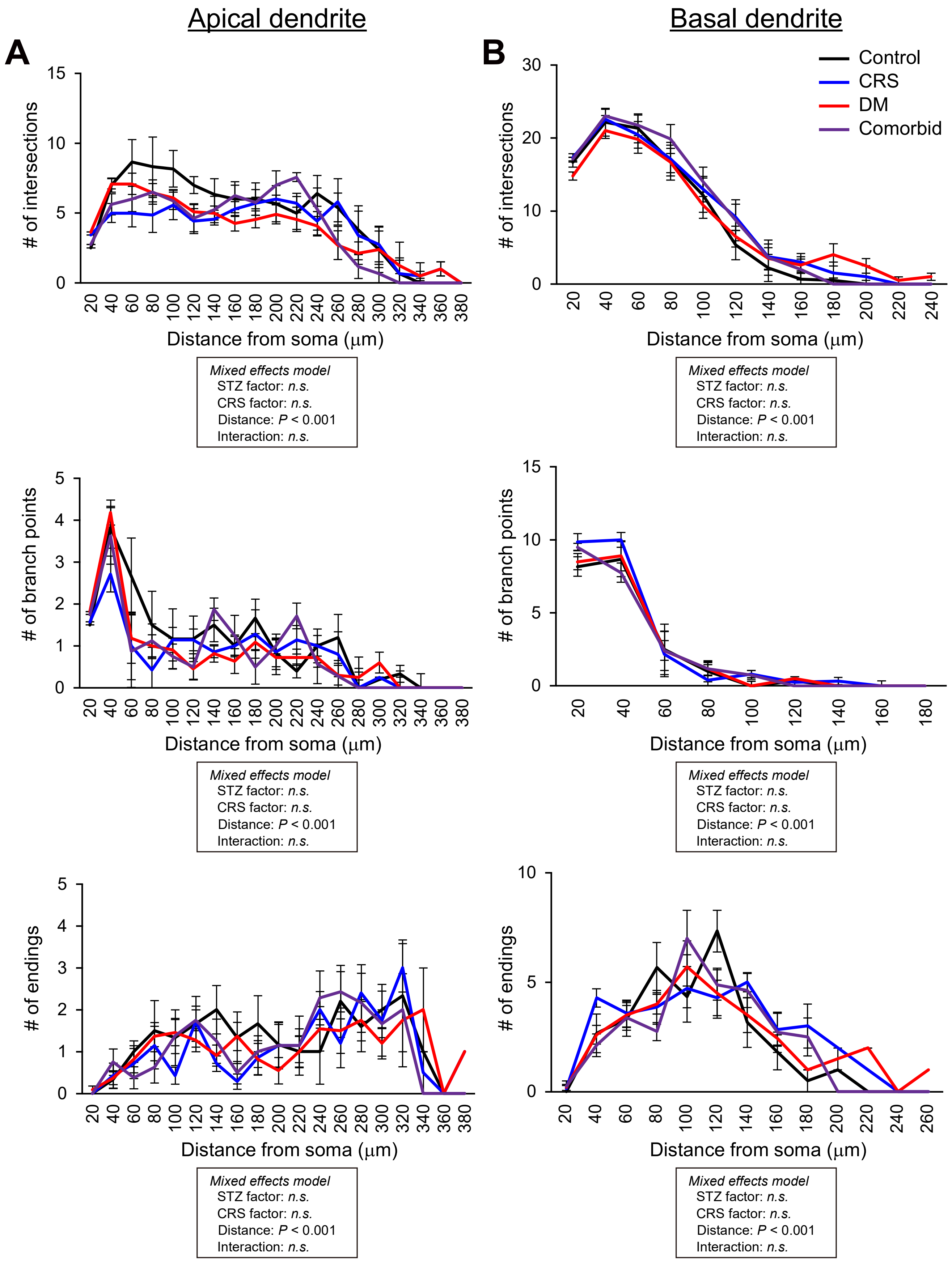

Supplementary figure 4 Sholl analysis of PFC neuron morphology. **(A)** Number of intersections (top), branch points (middle), and dendrite terminals (bottom) of apical dendrites. Control group, n = 6; CRS group, n = 7; DM group, n = 11; Comorbid group, n = 8. **(B)** Number of intersections (top), branch points (middle), and dendrite terminals (bottom) of basal dendrites. Control group, n = 5; CRS group, n = 7; DM group, n = 10; Comorbid group, n = 8; *n.s.*, not significant. Data are expressed as mean ± SEM.

Supplementary figure 5

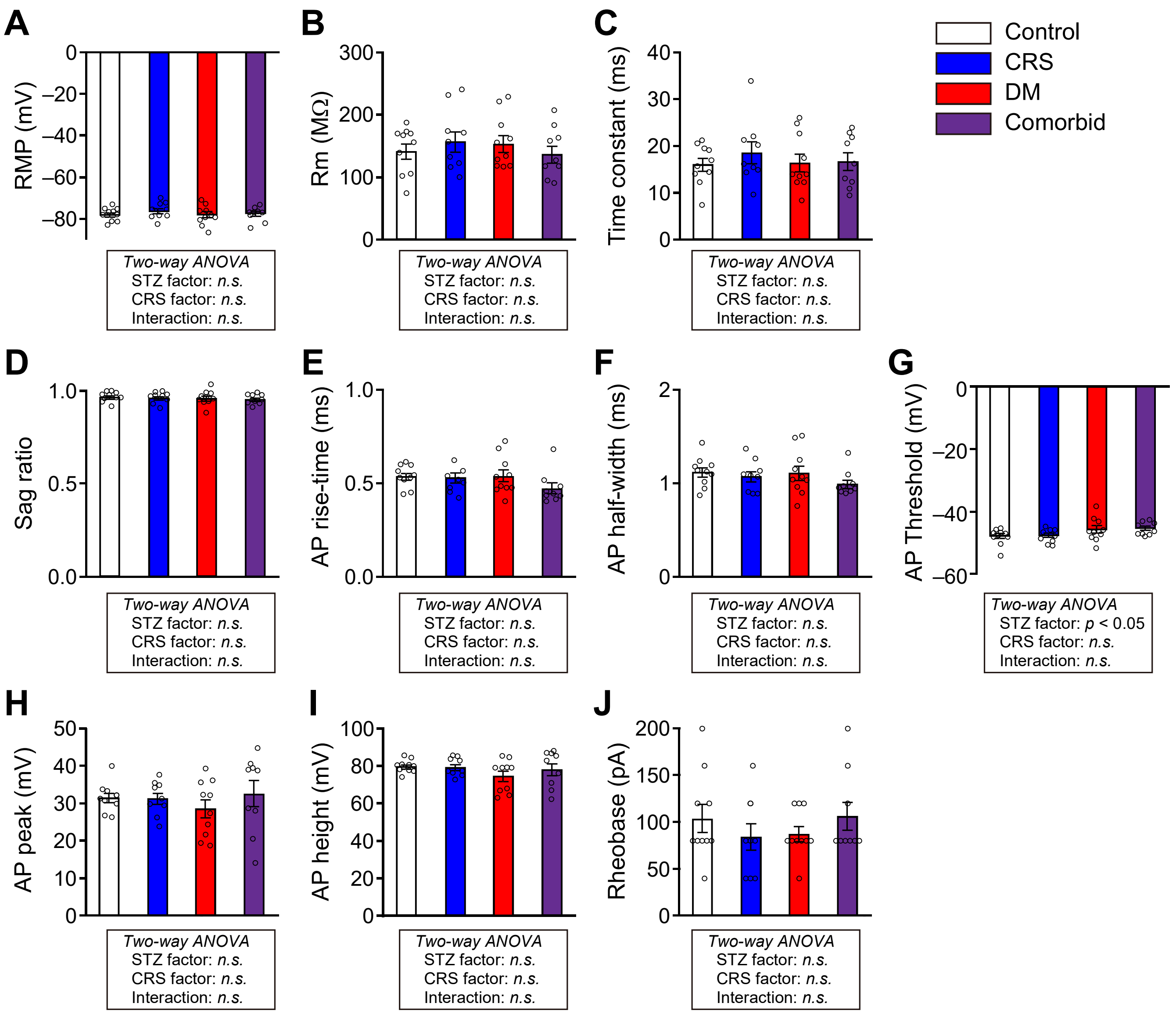

Supplementary figure 5 Electrophysiological characterization of layer II/III pyramidal neurons in the medial PFC. Membrane properties including **(A)** Resting membrane potential, **(B)** Membrane resistance (Rm), **(C)** Time constant, **(D)** Sag ratio, **(E)** Action potential (AP) rise-time, **(F)** AP half-width, **(G)** AP threshold, **(H)** AP peak, **(I)** AP height, and **(J)** Rheobase. Data are expressed as mean ± SEM. Control group, n = 10; CRS group, n = 9; DM group, n = 10; Comorbid group, n = 9; *n.s.*, not significant.
